# Dropfoot Following Alcoholic Paralylis

**Published:** 1899-03

**Authors:** 


### DROPFOOT FOLLOWING ALCOHOLIC PARALYLIS.

Fernand Gaucher draws attention (*These de Paris*, 1898) to the great importance of preventing permanent deformity in cases of alcoholic paralysis. The writer points out that in the large majority of cases permanent dropfoot and other deformities are due to the want of sufficient care on the part of the medical attendant. In all cases of alcoholic paralysis the antero-external group of muscles of the leg are early affected, allowing of dropfoot with internal rotation; consequently, if the patient is lying in bed, pressure of the bed covering tends to aggravate this pernicious position. It is important, therefore, to correct this deformity by means of suitable apparatus. This, on the other hand, requires considerable caution, as the low trophic condition of the tissues is liable to result in pressure lesions, should any of the different forms of rigid apparatus be employed. The writer therefore suggests using a simple wooden stand, thickly padded so as to maintain the foot at right angles to the leg. The next point is to obviate the pressure on the limbs by using a cradle. During the earlier part of the disease this should suffice, but so soon as the patient is able to bear it, passive movements and massage should be regularly employed, with a view to keeping the mobility of the joints in a healthy condition, and as quickly as possible to promote the nutrition of the wasted muscles. From an early period in the case the exhibition of galvanic currents is necessary. To this may be added the faradic so soon as the patient can stand it. In those cases where dropfoot becomes permanent, there is generally chronic myositis, and it is to prevent this condition that our best efforts should be employed. Sulphur baths, hot-air baths, and other methods have often been of extremely beneficial influence in these cases.—*British Medical Journal.*

